# Satisfaction of caregivers and older adults who use the Better at Home Program

**DOI:** 10.11606/s1518-8787.2022056003759

**Published:** 2022-04-27

**Authors:** Natália Romana Gomes da Silva, Garibaldi Dantas Gurgel, Domício Aurelio de Sá, Vanessa de Lima Silva, Rafael da Silveira Moreira

**Affiliations:** I Universidade Federal de Pernambuco Programa de Pós-Graduação em Gerontologia Recife PE Brasil Universidade Federal de Pernambuco. Programa de Pós-Graduação em Gerontologia. Recife, PE, Brasil; II Fundação Oswaldo Cruz Instituto Aggeu Magalhães Departamento de Saúde Coletiva Recife PE Brasil Fundação Oswaldo Cruz. Instituto Aggeu Magalhães. Departamento de Saúde Coletiva. Recife, PE, Brasil; III Universidade Federal de Pernambuco Departamento de Fonoaudiologia Recife PE Brasil Universidade Federal de Pernambuco. Departamento de Fonoaudiologia.Recife, PE, Brasil; IV Universidade Federal de Pernambuco Centro de Ciências Médicas Departamento de Saúde Coletiva Recife PE Brasil Universidade Federal de Pernambuco. Centro de Ciências Médicas. Departamento de Saúde Coletiva. Recife, PE, Brasil

**Keywords:** Caregivers, psychology, Job Satisfaction, Home Nursing, Health Services for the Aged, Patient Satisfaction

## Abstract

**OBJECTIVE:**

Evaluate the satisfaction of caregivers and older adults who use the home care service provided by the Better at Home Program (*Programa Melhor em Casa*) and its associated factors.

**METHODS:**

This is a cross-sectional study with data from the national survey on the Better at Home Program, conducted by the SUS General Ombudsman. We interviewed 5,595 caregivers and 950 older adults. We combined four questions related to satisfaction to formulate the dependent variable by means of latent class analysis and used 13 questions as independent variables.

**RESULTS:**

93.7% of the older adults and 90.2% of the caregivers were satisfied with the service. In the multiple analysis, the variables showing association with satisfaction were: also being accompanied by a family health team (older adults: OR = 4.22; p = 0.014), shorter time between referral and the first visit (older adults: OR = 10.20; p = 0.006), (caregiver: OR = 5.84; p < 0.001), taking examinations with medical requirement (older adults: OR = 5.74; p = 0.037), (caregiver: OR = 7.41; p < 0.001), consultation with specialist (caregiver: OR = 6.02; p < 0.001), visits out of the schedule when necessary (older adults: OR = 8.09; p = 0.014), (caregiver: OR = 1.81; p = 0.015) and understanding the orientations provided by the program team (caregiver: OR = 10.61; p < 0.001).

**CONCLUSIONS:**

The majority of the older adults and caregivers showed satisfaction with the program, with a distinction in the aspects associated with this satisfaction, in which only the characteristics directly related to the program influenced the satisfaction, while the same did not occur with the socioeconomic and demographic characteristics.

## INTRODUCTION

With the changes brought about by demographic and epidemiological transitions, the aging population has been growing quickly in the country^1,2^. In 2018, according to the Brazilian Institute of Geography and Statistics (IBGE), Brazilians’ life expectancy rose to 76.3 years of age and the older adults now represent 9.2% of the population^3^. Due to these changes, it is necessary to develop public and health policies that adequately meet the demand of this population^4^.

Home care emerges in Brazil as one of the tools for change and adaptation of care. It is a new type of health care that substitutes or complements the existing ones, characterized by a set of actions for health promotion, disease prevention and treatment, and rehabilitation provided at home, with a guarantee of continuity of care^5^.

Within the perspective of home care, in 2011, the federal government launched the *Programa Melhor em Casa* (PMC – Better at Home Program), indicated for people who have permanent or temporary difficulties in leaving their homes to get to the care center or for individuals who are in situations in which home care is the most appropriate for their treatment^6^.

The older adults indicated to PMC already present some type of pathology and/or limitations. Considering that, with advancing age, individuals can gradually weaken due to physiological and cognitive changes, limiting their functions and possibly causing functional dependence, the presence of a responsible caregiver identified is necessary^7,8^.

Regarding home care, on the one hand, the caregiver becomes the link between the team and the patient, being the main responsible for receiving the information made available by the health team, for the execution of the offered orientations, and for the care of the older patient. On the other hand, the teams are also responsible for the caregivers, facilitating the care process and offering support to their demands, listening to their doubts, promoting spaces for care and for the exchange of experiences among them, and promoting permanent education and training^7^.

The Better at Home Program, as a new national strategy for the Unified Health System, is fundamental for the planning and management of the system and for the implementation of evaluation processes of the services provided; with the objective of reordering the execution of actions and services, resizing them to meet the needs of the population, rationalizing the use of its resources^9^.

Thus, the Ministry of Health, via SUS General Ombudsman, conducted a national survey with the objective of evaluating the satisfaction of caregivers and older adults who use the home care service, provided by the Better at Home Program, and we describe the results of this analysis in this article.

## METHODS

This is a cross-sectional study, based on secondary data from the national survey on the Better at Home Program, conducted by the research center (NUPE – *núcleo de pesquisa*), of the SUS General Ombudsman (OuvSUS – Ouvidoria Geral do SUS).

The population was made up by two groups. One group was composed of caregivers of older adults, assisted by the Better at Home Program (PMC – *Programa Melhor em Casa*), of both genders and over 18 years old. And the other group was made up of older adults aged 60 years or more, users of the PMC and of both genders. Caregivers and older adults participated in the SUS General Ombudsman’s survey about the PMC.

The baseline survey was executed by the SUS General Ombudsman, with users of the PMC from all over Brazil, in the period from April to June 2017, as follows: we initially conducted it based on a registry containing 58,392 program users, originating from the e-SUS home care database, allocated in the e-SUS of primary care. After undergoing a cleanup of invalid, duplicate phone numbers and needed information that was not filled in, we had a reduction to 29,682 users, and of these, 6,545 (22%) were included in the eligibility criteria for the survey and answered the questionnaire, 5,595 caregivers and 950 older adults.

The survey was based on a questionnaire consisting of 22 questions, three related to the patient’s profile, one about the caregiver – when the caregiver answered the survey due to the patient’s inability to do it – and 18 questions about the Better at Home Program.

We applied the questionnaire via telephone contact by call center agents, monitored and supervised by a team of professionals from a call center company under the guidance and supervision of the SUS General Ombudsman.

We analyzed the dependent variable by means of latent class analysis (ACL – *análises de classes latentes*). This analysis becomes useful when more than one question contained in the questionnaire can measure, directly or indirectly, the satisfaction of users of the PMC, proving to be the best option to evaluate the dependent variable of this study, because the latent class aims to categorize individuals into classes, using observed items, and identify the questions that best distinguish the groups based on response patterns^10^.

Thus, we considered satisfaction as a latent phenomenon (not directly observed), but indirectly measured by questions directly developed and carried out using the questionnaire. We used the following questions: Are you satisfied with the service provided by the home care team?; How do you rate the care provided by the home care team?; Would you recommend the home care service to a friend or family member?; and In relation to the home care service, do you think anything needs to be improved?

Based on the answers to the four questions in the questionnaire, we identified the behavior of individuals, who were classified according to their performance. Thus, we created the latent variable, in which individuals were classified according to the probability of being in a specific class. In the construction of the latent variable, we created and tested several models with different class numbers until we found the ideal model to express the satisfaction variable. Thus, the individuals were classified into different groups, in which the intragroup characteristics showed homogeneity. However, in the intergroups the characteristics showed heterogeneity.

In choosing the best statistical model for the latent variable, we observed the following criteria: Akaike information criterion (AIC), Bayesian information criterion (BIC), adjusted Bayesian information criterion (adjusted BIC), always observing the lowest values when comparing the current model with the previous one. We also considered the highest entropy value and p-values < 0.05 in the likelihood ratio tests (LRT Vuong-Lo-Mendell-Rubin; LRT Lo-Mendell-Rubin; LRT Parametric Bootstrap)^10^.

The independent variables were: age, gender, schooling, family income, type of relationship between the caregiver and the older adult, region of the country where they live, knowledge regarding the SUS program, care shared with the family health team, time until receiving the first visit, frequency of visits, access to examinations and specialists when required, access to care provided by the program out of visiting hours, understanding the orientations given by the team’s professionals, invitation or participation in a course/group for caregivers offered by the team. All independent variables were defined based on the information contained in the questionnaires.

After creating the latent variable “satisfaction” (dependent variable), we performed Pearson’s chi-square test and analysis of the standardized residuals for the association measures between the dependent and independent variables. In cases where the test suppositions were not met, we applied Fisher’s exact test. All conclusions were drawn considering a significance level of 5%.

We used odds ratios (OR) and their respective 95% confidence intervals as measures of effect, by means of multinomial logistic regression models. Simple and multiple analyses were performed. In the simple analysis, the variables that obtained a p-value < 0.2511 were elective for the multiple analysis. For the multiple regression, we used the forward method and the we made the remaining conclusions with a significance level of 5%.

Statistical analysis was performed using the Mplus 8 program for the FTA and the statistical package for the social sciences (SPSS), version 20, for the other analyses.

The project is the result of a partnership developed between the Aggeu Magalhães Institute (IAM/Fiocruz-PE) and the Ministry of Health, via the SUS General Ombudsman (TED 18/2017), and was approved by the ethics and human research committee of the IAM/Fiocruz-PE, registered under CAAE number 25806419.9.0000.5190, according to resolution 466/12 on research developed with human beings.

## RESULTS

The analyses of the results were based on the answers to the questionnaire, in which 6,545 people participated, of which 5,595 (85.5%) were caregivers and 950 (14.5%) were older adults who were users, and, for a better understanding of these distinct groups, we analyzed them separately.

In the group of older adults who used the PMC, we found that the majority lived in the Southeast region (76.2%), were women (59.3%), aged between 60 and 70 years (52.5%), and had a family income of up to two minimum wages (85.9%). The youngest old adult was 60 years old and the oldest was 99 years old, with an average age of 70 years. In the caregivers group, we found that the majority lived in the Southeast region (72%), they cared for older adults who were women (59.1%), aged between 60 and 81 years (53%), with the youngest aged 60 years and the oldest aged 109 years, with a median of 81 years, and had a family income of one to two minimum wages (74.5%). The caregivers had complete or incomplete high school education (40.2%) and were classified as informal caregivers (97.1%).

Regarding the description of the PMC, in the group of the older adults, we found that the majority knew it was a SUS program (73.1%). Besides the PMC team they were also followed by the family health team (58.3%), waited three days to start being assisted by the program (34.7%), receiving visits from the team daily or weekly (63.9%), could take examinations and consultations with specialists all the times they were required by the physicians (55.5% and 40.5%), visits out of scheduled appointments when necessary (53.9%), and always understood the orientations given by the team professionals about their care (84.1%).

In the caregivers group, for this same item, PMC description, we found that most knew was a SUS program (85.6%), besides the PMC team they were also followed by the family health team (53.6%), waited three to seven days to start being assisted by the program (27.4%), received visits from the team weekly (46.6%), could take examinations and consultations with specialists for the older adults all the times they were required by the physicians (58.3% and 40.3%), receive visits out of scheduled appointments when necessary (60.2%), always understood the orientations given by the team professionals about their care (90.6%), and were not invited or participated in courses for caregivers (80.2%).

To verify the prevalence of satisfaction, both for older adults and caregivers, five models were tested in the latent class analysis (LCA). The model with the highest parsimony was the one with three classes, both for caregivers and the older adults, as they presented high entropy value together with significant LRT values, besides presenting lower BIC and AIC values.

The nomenclature of the classes was assigned according to the response patterns. The class named “satisfied” showed positive responses on all questions. The “moderately satisfied” class showed mixed response patterns in which participants were satisfied with the service and would recommend it to others, but believed that something could still be improved and rated the service as regular. The class named “not satisfied” presented negative answers to all questions. For this analysis, the answer option “don’t know/no answer” was considered as missing value. Thus, we considered the answers of 26 older adults and 129 caregivers as missing. Both the older adults and the caregivers were mostly satisfied with the PMC, representing 93.7% and 90.2%, respectively (Table 1).

**Table 1:** Absolute and relative frequencies of the classes and their respective names in the populations of older adults and caregivers.

Classification	Older adults n (%)	Caregivers n (%)
Not satisfied	25 (2.7)	194 (3.5)
Moderately satisfied	33 (3.6)	340 (6.2)
Satisfied	866 (93.7)	4,932 (90.2)

When performing the simple analysis in each group, we observed some distinctions. In the group of older adults, only the variables gender, family income, and knowing the program is from SUS were not elective for multiple analysis (Table 2). When performing the multiple analysis, the following variables remained associated with the satisfaction of the older adults: the fact that they were also accompanied by a family health team, the shorter time between referral and the first visit by the PMC, take examinations when required by the physician, and visits out of scheduled appointments when necessary (Table 3).

**Table 2:** Simple analysis of older adults’ satisfaction with respect to socioeconomic, demographic, and PMC-related variables.

Socioeconomic, demographic, and PMC-related factors	Satisfaction of the older adults					
	Satisfied			Moderately satisfied		
	OR	95%CI	p	OR	95%CI	p
Gender						
Woman	1.33	0.60–2.96	0.477	1.42	0.49–4.06	0.513
Man	1.00			1.00		
Age						
≤ median (70 years old)	0.89	0.40–1.98	0.776	0.51	0.17–1.46	0.212
> median (70 years old)	1.00			1.00		
Income						
0 to 2 minimum wages	0.81	0.23–2.74	0.736	1.27	0.23–6.93	0.780
More than 2 minimum wages	1.00			1.00		
Knowing the program is from SUS						
Yes	1.57	0.68–362	0.281	1.50	0.49–4.59	0.478
No	1.00			1.00		
Care shared with the family health team						
Yes	3.78	1.56–9.16	0.003	2.42	0.79–7.33	0.118
No	1.00			1.00		
Time between referral and receiving the first visit						
Up to 3 days	17.36	3.66–82.37	< 0.001	3.21	0.47–21.80	0.232
From 3 to 7 days	11.81	2.48–56.15	0.002	4.50	0.70–28.79	0.112
From 8 to 15 days	6.28	1.64–23.99	0.007	1.28	0.19–8.43	0.793
From 16 to 30 days	2.53	0.74–8.61	0.137	0.32	0.02–3.55	0.355
More than 30 days	1.00			1.00		
Frequency of visits						
Daily or Weekly (once a week or more)	14.59	4.36–48.77	< 0.001	3.85	0.85–17.32	0.078
Once a month	6.28	1.64–23.99	0.007	1.28	0.19–8.43	0.793
2 times a month	2.53	0.74–8.61	0.137	0.32	0.29–3.55	0.355
3 times a month	1.00			1.00		
Access to examinations, when required						
Yes, every time	16.53	5.02–54.35	< 0.001	4.20	0.83–21.04	0.081
Yes, sometimes	2.64	0.90–7.73	0.075	2.40	0.52–11.10	0.260
No, did not need to take any examination	6.36	1.92–21.03	0.002	2.10	0.38–11.58	0.395
2No, could not take any examination	1.00			1.00		
Access to consultation with specialists, when required						
Yes, every time	16.64	4.59–60.25	< 0.001	2.80	0.57–13.75	0.205
Yes, sometimes	3.72	1.16–11.95	0.027	2.70	0.63–11.46	0.178
No, consultation with a specialist were not required	6.75	2.46–18.53	< 0.001	1.20	0.29–4.90	0.800
No, could not take any consultation	1.00			1.00		
Visit by PMC team out of scheduled appointments						
Yes	4.32	1.39–13.37	0.011	4.33	1.12–16.67	0.033
No	0.14	0.05–0.36	< 0.001	0.40	0.08–1.89	0.252
There was no need	1.00			1.00		
Understand the care orientations given by the professionals of the PMC team						
Always	10.16	2.04–50.45	0.005	0.94	0.13–6.54	0.954
Most of the time	2.88	0.52–15.94	0.224	1.11	0.14–8.68	0.920
Almost never	0.16	0.01–1.96	0.154	0.33	0.01–6.65	0.472
Never	1.00			1.00		

PMC: Better at Home Program (*Programa Melhor em Casa*); SUS: Unified Health System (*Sistema Único de Saúde*); OR: odds ratio; 95%CI: 95% confidence interval.

Note: the category “not satisfied” was used as a reference for the analysis.

**Table 3:** Multiple analysis of older adults’ satisfaction with respect to socioeconomic, demographic, and PMC-related variables.

Socioeconomic, demographic, and PMC-related factors	Satisfaction of the older adults					
	Satisfied			Moderately satisfied		
	OR	95%CI	p	OR	95%CI	p
Care shared with the family health team						
Yes	4.22	1.33–13.34	0.014	3.43	0.83–14.16	0.088
No	1.00			1.00		
Time between referral and receiving the first visit						
Up to 3 days	10.20	1.95–53.23	0.006	2.07	0.28–15.41	0.474
From 3 to 7 days	9.66	1.80–51.78	0.008	3.30	0.45–24.01	0.237
From 8 to 15 days	6.45	1.44–28.75	0.014	1.26	0.17–9.24	0.820
From 16 to 30 days	1.57	0.40–6.15	0.515	0.20	0.01–2.45	0.210
More than 30 days	1.00			1.00		
Access to examinations, when required						
Yes, every time	5.74	1.11–29.77	0.037	1.53	0.19–12.33	0.685
Yes, sometimes	0.56	0.11–2.77	0.479	0.44	0.05–3.48	0.441
No, did not need to take any examination	1.57	0.31–7.94	0.584	0.12	0.008–1.93	0.136
No, could not take any examination	1.00			1.00		
Visit by PMC team out of scheduled appointments						
Yes	4.96	1.23–19.89	0.024	8.09	1.52–43.05	0.014
No	0.22	0.06–0.78	0.019	0.24	0.02–2.69	0.248
There was no need	1.00			1.00		

PMC: Better at Home Program (*Programa Melhor em Casa*); OR: odds ratio; 95%CI: 95% confidence interval.

Note: the category “not satisfied” was used as a reference for the analysis.

In the simple analysis, performed with the caregivers, we observed that only the variables gender and age were not elective for the multiple analysis (Table 4). After the multiple analysis, the following variables remained associated with caregiver satisfaction: the shortest time between referral and the first visit by the PMC, take examinations and consultations with specialists when required by the physician, visits out of scheduled appointments when necessary, and understanding the orientations given by the program team (Table 5).

**Table 4:** Simple analysis of caregiver satisfaction with respect to socioeconomic, demographic, and PMC-related variables.

Socioeconomic, demographic, and PMC-related factors	Satisfaction of the caregivers					
	Satisfied			Moderately satisfied		
	OR	95%CI	p	OR	95%CI	p
Gender of the older adult						
Woman	0.92	0.69–1.24	0.623	1.10	0.77–1.59	0.582
Man	1.00			1.00		
Age of the older adult						
≤ median (81 years old)	0.91	0.68–122	0.565	1.17	0.82–1.67	0.382
> median (81 years old)	1.00			1.00		
Family income of the older adult						
0 to 1 minimum wage	0.94	0.47–1.86	0.870	1.86	0.82–4.23	0.137
Between 1 and 2 minimum wages	1.23	0.85–1.76	0.257	1.58	0.99–2.52	0.055
More than 2 minimum wages	1.00			1.00		
Schooling						
Literate	0.38	0.04–3.44	0.389	0.75	0.06–8.38	0.815
Elementary school (complete or incomplete)	0.29	0.04–2.11	0.223	0.38	0.04–3.35	0.387
High school (complete or incomplete)	0.21	0.03–1.59	0.134	0.34	0.04–3.00	0.335
Higher Education (complete or incomplete)	0.21	0.02–1.59	0.134	0.31	0.03–2.78	0.297
Postgraduate	0.05	0.00–0.45	0.008	0.16	0.01–1.98	0.154
Illiterate	1.00			1.00		
Degree of relationship with the patient						
Informal	0.16	0.02–1.19	0.074	0.34	0.04–3.00	0.337
Formal	1.00			1.00		
knowing the program is from SUS						
Yes	1.41	0.97–2.04	0.068	1.10	0.70–1.75	0.662
No	1.00			1.00		
Care shared with the family health team						
Yes	2.24	1.66–3.03	< 0.001	1.23	0.85–1.78	0.258
No	1.00			1.00		
Time between referral and receiving the first visit						
Up to 3 days	10.48	5.81–18.91	< 0.001	2.18	1.09–4.36	0.026
From 3 to 7 days	9.11	5.27–15.75	< 0.001	2.24	1.18–4.25	0.013
From 8 to 15 days	3.59	2.30–5.60	< 0.001	1.44	0.83–2.49	0.184
From 16 to 30 days	1.59	1.04–2.42	0.032	1.04	0.61–1.77	0.880
More than 30 days	1.00			1.00		
Frequency of visits						
Daily	2.27	0.60–2.57	0.225	0.97	0.20–4.76	0.978
Weekly (once a week or more)	1.54	0.54–4.39	0.415	1.33	0.38–4.59	0.648
Once a month	0.33	0.12–0.93	0.036	0.58	0.17–1.96	0.384
2 times a month	0.62	0.21–1.80	0.385	0.74	0.21–2.64	0.650
3 times a month	1.00			1.00		
Access to examinations, when required						
Yes, every time	21.04	13.95–31.73	< 0.001	4.78	2.85–8.03	< 0.001
Yes, sometimes	4.99	3.34–7.45	< 0.001	3.19	1.90–5.35	< 0.001
No, did not need to take any examination	6.04	3.95–9.24	< 0.001	3.34	1.94–5.74	< 0.001
No, could not take any examination	1.00			1.00		
Access to consultation with specialists, when required						
Yes, every time	18.86	11.06–32.15	< 0.001	2.90	1.58–5.32	0.001
Yes, sometimes	3.01	1.96–4.62	< 0.001	1.28	0.76–2.17	0.344
No, consultation with a specialist were not required	3.89	2.75–5.50	< 0.001	1.22	0.80–1.88	0.350
No, could not take any consultation	1.00			1.00		
Visit by PMC team out of scheduled appointments						
Yes	2.51	1.70–3.68	< 0.001	1.37	0.87–2.16	0.171
No	0.09	0.06–0.13	< 0.001	0.51	0.33–0.79	0.003
There was no need	1.00			1.00		
Understand the care orientations given by the professionals of the PMC team						
Always	52.94	29.13–96.19	< 0.001	6.38	3.01–13.52	< 0.001
Most of the time	25.96	12.64–53.31	< 0.001	6.35	2.62–15.39	< 0.001
Almost never and Never	1.00			1.00		
Invitation and/or participation in a course/group for caregivers offered by the team						
Yes	2.60	1.59–4.25	< 0.001	1.30	0.72–2.34	0.376
No	1.00			1.00		

PMC: Better at Home Program (*Programa Melhor em Casa*); SUS: Unified Health System (*Sistema Único de Saúde*); OR: odds ratio; 95%CI: 95% confidence interval.

Note: the category “not satisfied” was used as a reference for the analysis.

**Table 5:** Multiple analysis of caregiver satisfaction with respect to socioeconomic, demographic, and PMC-related variables.

Socioeconomic, demographic, and PMC-related factors	Satisfaction of the caregivers					
	Satisfied			Moderately satisfied		
	OR	95%CI	p	OR	95%CI	p
Time between referral and receiving the first visit						
Up to 3 days	5.84	2.91–11.75	< 0.001	1.76	0.81–3.79	0.149
From 3 to 7 days	5.40	2.88–10.12	< 0.001	1.67	0.83–3.37	0.146
From 8 to 15 days	3.28	1.87–5.76	< 0.001	1.40	0.75–2.63	0.282
From 16 to 30 days	1.18	0.71–1.99	0.510	0.81	0.45–1.45	0.481
More than 30 days	1.00			1.00		
Access to examinations, when required						
Yes, every time	7.41	4.17–13.14	< 0.001	4.31	2.22–8.37	< 0.001
Yes, sometimes	2.97	1.74–5.06	< 0.001	2.79	1.50–5.18	0.001
No, did not need to take any examination	2.83	1.55–5.14	0.001	3.58	1.79–7.16	< 0.001
No, could not take any examination	1.00			1.00		
Access to consultation with specialists, when required						
Yes, every time	6.02	2.89–12.55	< 0.001	1.72	0.78–3.78	0.176
Yes, sometimes	1.32	0.75–2.31	0.330	0.68	0.36–1.27	0.231
No, consultation with a specialist were not required	1.68	1.01–2.80	0.044	0.63	0.36–1.13	0.126
No, could not take any consultation	1.00			1.00		
Visit by PMC team out of scheduled appointments						
Yes	1.81	1.12–2.91	0.015	1.21	0.70–2.07	0.488
No	0.14	0.09–0.24	< 0.001	0.67	0.39–1.14	0.142
There was no need	1.00			1.00		
Understand the care orientations given by the professionals of the PMC team						
Always	10.61	4.27–26.37	< 0.001	3.16	1.31–7.64	0.010
Most of the time	7.01	2.47–19.85	< 0.001	3.49	1.24–9.85	0.018
Almost never and Never	1.00				1.00	

PMC: Better at Home Program (*Programa Melhor em Casa*); OR: odds ratio; 95%CI: 95% confidence interval.

Note: the category “not satisfied” was used as a reference for the analysis.

The results of the analysis of the independent variables that showed association with the older adults and caregivers group were similar, that is, practically equal factors seem to influence satisfaction, and none of them were related to socioeconomic and demographic factors. However, the variable region was not put for analysis in the regressions, since it did not present enough individuals in the subgroups for the analysis to be performed. There was a large concentration of satisfied older adults and caregivers who lived in the Southeast.

## DISCUSSION

A profile of caregivers and older adults served by the Better at Home Program was similar to other home care programs in terms of socioeconomic and demographic characteristics^12,13^. The PMC analyzed by this research presented some characteristics that corroborate what is recommended by the program’s legislation and others that did not present similarities^14^.

Regarding satisfaction, the present study found high levels both in the group of the older adults (93.7%) and in the group of the caregivers (90.2%). Studies have often shown high levels of user satisfaction internationally and in Brazil, and this occurs regardless of the characteristic of the service and the methodology used^15^. Some researchers attribute this to the gratitude bias, where there is a reluctance to express negative opinions^18^, and politeness norms, such as social obligations to show respect for authorities or understanding criticism as something that shows social inconvenience, and this can also be associated with high satisfaction^19^.

However, it is important to emphasize the relevance of evaluating the satisfaction of a health service as a form of subsidy for planning and feedback on the actions implemented, in addition to empowering the user within the construction of the health service. Furthermore, in an attempt to reduce some bias, this study evaluated satisfaction by means of latent class analysis, because it considered its complexity and the difficulty in measuring phenomena such as satisfaction. Thus, the satisfaction of the older adults and caregivers with the PMC can also be justified by the quality of the services offered, since in its characterization the program showed positive indicators in practically all the aspects evaluated.

We also found factors associated with this high satisfaction, both for the older adults and for the caregivers. Regarding these factors, the older adults’ group and the caregivers’ group presented similar results after multiple analysis.

Martins et al.^20^ (2014) report that according to the perception of users, the factors that can ensure a better quality of public health services, in all complexities, include reducing the waiting time and ease of scheduling appointments in specialized services, corroborating the results of this study. Quality as measured by user perception is based on the user’s concept of whether a service meets or fails to meet care needs^17,21^. That is, users make comparisons between the performance of services and their expectations. The results are good when the perceived quality is obtained^22,23^.

In this study, the waiting time to start the PMC services was rated as short, mostly reaching a maximum of seven days in the caregivers’ group, and approximately six times more likely to be satisfied with the service if this time was reached. In the group of older adults this time reached three days, increasing by approximately 10 times the chance of satisfaction with the service. Thus, the expectation was reached and consequently the performance of the service and its quality obtained good results, making the older adults and the caregivers satisfied with the program.

Regarding the guarantee of access to services of different complexities, health care networks have been created providing the possibility of continuous and integral attention to users^24^. The home care service is part of the health care network and its objective is to articulate with other health care points, mainly hospitals, emergency services, and primary care, seeking to avoid direct users’ demands. It must also ensure referral flows to specialties and for complementary diagnostic methods, both for elective and emergency situations^7^. As the teams are often located in hospitals or emergency care units, the ease of access of their users to other services may increase, which does not occur in other public health services^25^.

Thus, the fact that the older adults and caregivers obtained access, most of the time, whenever they were asked, showed a significant relationship and led to an approximately up to seven-fold increase in the chance of being satisfied with the PMC.

The visits out of scheduled appointments also presented a significant association in both groups (older adults and caregivers), with those who received these visits being more satisfied with the program, showing a greater availability of the team for care. A study conducted by Gorina et al.^26^ (2014) analyzed two models of home care, in which the difference between them was in the time provided by the team for the care. The model in which the team provided more time with the patients showed better levels of satisfaction from the users, compared to the other model, in which the team provided less time. Arruda and Bosi^25^ (2017) place the availability of the team as an offer of help, highlighting the need for the establishment of a bond, which presents itself as a determinant regarding the satisfaction of users in relation to care.

Regarding the understanding of the guidelines informed by the team, a study by Kajonius and Kazemi^27^ (2016) found a strong association between satisfaction and access to information by users. Sharing information involves them in an active partnership in care. Factors like this, involving the relationship between the team and users or caregivers, seems to be essential in the satisfaction with the service. In this study, understanding this information/guidance was a strong factor among caregivers in influencing satisfaction, leading to a 10 times greater chance of satisfaction among those who always understood compared to those who never or almost never understood them.

Finally, the older adults being accompanied by the PMC and the family health team was associated with satisfaction, that is, those who had their care shared were more satisfied. The sharing of care is related to the team’s understanding on the patient’s needs and sensitivity to the specificities of each user. Promoting this shared care with the teams or services that make up the network is essential, it increases the effectiveness and efficiency of the health care network and, as the management of patient care in home care is complex, there is a need for integration of considerable part of the network^28^. Possibly, within this perspective of shared care, older adults feel more secure and comfortable with the care and, consequently, this reflects in the perception of the quality of the service and in their satisfaction.

In summary, in both groups (older adults and caregivers) more than 90% of the participants showed satisfaction with the program. And among the investigated factors, no socioeconomic or demographic variable remained associated with satisfaction. While the variables related to the program, such as: time between referral and first visit, access to examinations and specialists, visits out of scheduled appointments, understanding of the orientations transmitted by the team, and being followed by the PMC and the Family Health team, showed a statistically significant association.

Therefore, studies like this one enable a better understanding about the main factors related to the satisfaction of users and their caregivers, helping in the comprehension regarding the program and, also, helping in the planning of health management.
