# Supplementary material for: Satisfaction of caregivers and older adults who use the Better at Home Program
Source: Rev Saude Publica. 2022 Apr 27;56:34. doi: 10.11606/s1518-8787.2022056003759 (PMC9060760; doi:10.11606/s1518-8787.2022056003759)
Supplement: Translation

# Translation: Satisfação dos cuidadores e dos idosos usuários do Programa Melhor em Casa

**Authors:** Natália Romana Gomes da Silva, Garibaldi Dantas Gurgel, Domício Aurelio de Sá, Vanessa de Lima Silva, Rafael da Silveira Moreira

**Affiliations:** I Brasil Universidade Federal de Pernambuco. Programa de Pós-Graduação em Gerontologia. Recife, PE, Brasil; II Brasil Fundação Oswaldo Cruz. Instituto Aggeu Magalhães. Departamento de Saúde Coletiva. Recife, PE, Brasil; III Brasil Universidade Federal de Pernambuco. Departamento de Fonoaudiologia. Recife, PE, Brasil; IV Brasil Universidade Federal de Pernambuco. Centro de Ciências Médicas. Departamento de Saúde Coletiva. Recife, PE, Brasil

**Keywords:** Cuidadores, psicologia, Satisfação no Emprego, Assistência Domiciliar, Serviços de Saúde para Idosos, Satisfação do Paciente

## Abstract

**OBJETIVO:**

Avaliar a satisfação dos cuidadores e dos idosos usuários do serviço de atenção domiciliar por meio do Programa Melhor em Casa e seus fatores associados.

**METÓDOS:**

Trata-se de um estudo transversal com dados da pesquisa nacional sobre o Programa Melhor em Casa, conduzida pela Ouvidoria Geral do SUS. Foram entrevistados 5.595 cuidadores e 950 idosos. Quatro perguntas relacionadas à satisfação foram unidas para formulação da variável dependente por meio da análise de classes latentes e 13 perguntas foram utilizadas como variáveis independentes.

**RESULTADOS:**

Mostraram-se satisfeitos com o serviço, 93,7% dos idosos e 90,2% dos cuidadores. Na análise múltipla, as variáveis que apresentaram associação com a satisfação foram: também ser acompanhados por uma equipe de saúde da família (idoso: OR = 4,22; p = 0,014), menor tempo entre o encaminhamento e a primeira visita (idoso: OR = 10,20; p = 0,006), (cuidador: OR = 5,84; p < 0,001), realização de exames quando havia solicitação médica (idoso: OR = 5,74; p = 0,037), (cuidador: OR = 7,41; p < 0,001), realização de consulta com especialista (cuidador: OR = 6,02; p < 0,001), ter visitas fora do horário agendado quando fosse necessário (idoso: OR = 8,09; p = 0,014), (cuidador: OR = 1,81; p = 0,015) e compreender as orientações que eram passadas pela equipe do programa (cuidador: OR = 10,61; p < 0,001).

**CONCLUSÕES:**

Constatou-se que a maioria dos idosos e dos cuidadores demonstraram satisfação com o programa, havendo distinção nos aspectos associados a essa satisfação, nos quais apenas as características diretamente relacionadas ao programa influenciaram na satisfação, não ocorrendo o mesmo com as características socioeconômicas e demográficas.

## INTRODUÇÃO

Com as alterações ocasionadas pelas transições demográficas e epidemiológicas, a população idosa vem crescendo de forma acelerada no país^1,2^. Em 2018, segundo o Instituto Brasileiro de Geografia e Estatística (IBGE), a expectativa de vida do brasileiro passou para 76,3 anos de idade e os idosos chegaram a representar 9,2% da população^3^. Devido a essas alterações, torna-se necessária a realização de políticas públicas e de saúde que atendam adequadamente à demanda dessa população^4^.

A atenção domiciliar surge no Brasil como uma das ferramentas de mudança e adequação do cuidado. Ela é uma nova modalidade de atenção à saúde substitutiva ou complementar às já existentes, caracterizada por um conjunto de ações de promoção à saúde, prevenção e tratamento de doenças e reabilitação prestadas em domicílio, com garantia de continuidade de cuidados^5^.

Dentro da perspectiva da atenção domiciliar, foi lançado pelo governo federal, em 2011, o Programa Melhor em Casa (PMC), indicado para as pessoas que apresentem dificuldades definitivas ou temporárias de sair de seu domicílio para chegar até o local de atendimento ou ainda para indivíduos que estejam em situações em que a atenção domiciliar é a mais indicada para seu tratamento^6^.

Os idosos indicados ao PMC já apresentam algum tipo de patologia e/ou limitações. Tendo em vista que, com o avanço da idade, os indivíduos podem debilitar-se gradativamente devido às alterações fisiológicas e cognitivas, limitando as suas funções e podendo ocasionar a dependência funcional, torna-se necessária a presença de um cuidador responsável identificado^7,8^.

No que tange à atenção domiciliar, por um lado, o cuidador torna-se o elo entre a equipe e o paciente, sendo ele o principal responsável pelo recebimento das informações disponibilizadas pela equipe de saúde, pela execução das orientações ofertadas e pelo cuidado ao paciente idoso. Por outro lado, as equipes também ficam responsáveis pelos cuidadores, facilitando o processo do cuidar e ofertando apoio às suas demandas, acolhendo suas dúvidas, promovendo espaços de cuidado e de troca de experiência entre eles e promovendo educação permanente e capacitação^7^.

O Programa Melhor em Casa, por ser uma nova estratégia nacional do Sistema Único de Saúde, torna-se fundamental, para o planejamento e gestão do programa, a instituição de processos avaliativos dos serviços prestados, com o objetivo de reordenar a execução das ações e serviços, redimensionando-os de forma a contemplar as necessidades da população, racionalizando o uso dos seus recursos^9^.

Nesse sentido, o Ministério da Saúde, por meio da Ouvidoria Geral do SUS, realizou uma pesquisa nacional com o objetivo de avaliar a satisfação dos cuidadores e dos idosos usuários do serviço de atenção domiciliar, no Programa Melhor em Casa, sendo os resultados dessas análises descritos neste artigo.

## MÉTODOS

Trata-se de um estudo transversal, baseado nos dados secundários da pesquisa nacional sobre o Programa Melhor em Casa, realizada pelo núcleo de pesquisa (NUPE), da Ouvidoria Geral do SUS (OuvSUS).

A população foi formada por dois grupos. Um grupo foi composto por cuidadores de idosos, atendidos pelo Programa Melhor em Casa, de ambos os sexos e com idade acima de 18 anos. E o outro grupo, por idosos com 60 anos de idade ou mais, usuários do PMC e de ambos os sexos. Cuidadores e idosos haviam participado da pesquisa base da Ouvidoria Geral do SUS, sobre o PMC.

A pesquisa base foi executada pela Ouvidoria Geral do SUS, com os usuários do PMC de todo Brasil, no período de abril a junho de 2017, da seguinte maneira: inicialmente foi realizada com base em um cadastro contendo 58.392 usuários do programa, originários do banco de dados do e-SUS atenção domiciliar, alocados no e-SUS da atenção básica. Depois de submetida a uma limpeza dos telefones inválidos, duplicados e de informações obrigatórias que não estavam preenchidas, houve uma redução para 29.682 usuários, e desses, 6.545 (22%) foram incluídos nos critérios de elegibilidade para a pesquisa e responderam ao questionário, sendo 5.595 cuidadores e 950 idosos.

A pesquisa foi feita a partir de um questionário composto de 22 perguntas, sendo três relacionadas ao perfil do paciente, uma sobre o cuidador – quando ele respondeu à pesquisa por impossibilidade do paciente de fazê-lo – e 18 questões relativas ao Programa Melhor em Casa.

O questionário foi aplicado por meio de contato telefônico por teleatendentes, monitorados e supervisionados por uma equipe de profissionais de uma empresa de teleatendimento sob a orientação e supervisão da Ouvidoria Geral do SUS.

A variável dependente foi analisada por meio de análises de classes latentes (ACL). Essa análise se torna útil na medida em que mais de uma pergunta contida no questionário se mostra capaz de mensurar, de maneira direta ou indireta, a satisfação dos usuários do PMC, mostrando-se a melhor opção para avaliar a variável dependente deste estudo, pois a classe latente tem como objetivo categorizar os indivíduos em classes, usando itens observados, e identificar as questões que melhor distinguem os grupos com base nos padrões de respostas^10^.

Sendo assim, a satisfação foi considerada um fenômeno latente (não observado diretamente), mas indiretamente mensurado por perguntas diretamente desenvolvidas e realizadas por meio do questionário. As perguntas utilizadas foram as seguintes: O(a) Sr.(a) está satisfeito com o serviço prestado pela equipe de atenção domiciliar?; Como o(a) Sr.(a) avalia o atendimento prestado pela equipe de atenção domiciliar?; O(a) Sr.(a) recomendaria o serviço de atenção domiciliar para um amigo ou familiar?; e Em relação ao serviço de atenção domiciliar, o(a) Sr.(a) acha que alguma(s) coisa(s) precisa(m) ser melhorada(s)?

Com base nas respostas das quatro perguntas do questionário foi identificado o comportamento dos indivíduos, que foram classificados quanto ao seu desempenho. Dessa forma, foi criada a variável latente, na qual os indivíduos foram classificados de acordo com a probabilidade de estar em uma determinada classe. Na construção da variável latente, vários modelos com diferentes números de classe foram criados e testados até se encontrar o modelo ideal para expressar a variável satisfação. Assim, os indivíduos foram classificados em distintos grupos, nos quais as características intragrupos apresentaram homogeneidades. No entanto, nos intergrupos as características mostraram heterogeneidade.

Na escolha do melhor modelo estatístico da variável latente, foram observados os seguintes critérios: critério de informação e Akaike (AIC), critério de informação Bayesiano (BIC), critério de informação Bayesiano ajustado (BIC ajustado), observando-se sempre os menores valores quando comparados o modelo atual com o anterior. Também foi considerado o maior valor de entropia e os valores de p < 0,05 nos testes de razão de verossimilhanças (LRT Vuong-Lo-Mendell-Rubin; LRT Lo-Mendell-Rubin; LRT Bootstrap paramétrico)^10^.

As variáveis independentes foram: idade, sexo, escolaridade, renda familiar, tipo de relação do cuidador com o idoso, região do país que residem, conhecimento que o programa é do SUS, atendimento compartilhado com a equipe de saúde da família, tempo até receber a primeira visita, frequência de atendimentos, acesso a exames e especialistas, quando solicitados, acesso a atendimento pelo programa fora do horário das visitas, compreensão das orientações realizadas pelos profissionais da equipe, convite ou participação de curso/grupo para cuidadores ofertados pela equipe. Todas as variáveis independentes foram definidas com base nas informações contidas nos questionários.

Após a criação da variável latente “satisfação” (variável dependente), foi realizado o teste de qui-quadrado de Pearson e análise dos resíduos padronizados para as medidas de associação entre a variável dependente e as independentes. Nos casos em que as suposições do teste não foram satisfeitas, aplicou-se o teste exato de Fisher. Todas as conclusões foram tiradas considerando o nível de significância de 5%.

Como medida de efeito foi utilizado o *odds ratio* (*OR*) e seus respectivos intervalos de confiança de 95%, por meio de modelos de regressão logística multinomial. Foram realizadas análises simples e múltipla. Na análise simples foram eletivas para a análise múltipla as variáveis que obtiveram valor-p < 0,25^11^. Para a regressão múltipla foi utilizado o método *forward* e as demais conclusões foram tomadas com nível de significância de 5%.

A análise estatística foi realizada por meio do programa Mplus 8 para a ACL e do programa *statistical package for the social sciences* (SPSS), versão 20, para as demais análises.

O projeto é fruto de uma parceria desenvolvida entre o Instituto Aggeu Magalhães (IAM/Fiocruz-PE) e o Ministério da Saúde, por meio da Ouvidoria Geral do SUS (TED 18/2017), e foi aprovado pelo comitê de ética e pesquisa com seres humanos do IAM/Fiocruz-PE, registrado sob o número CAAE 25806419.9.0000.5190, de acordo com a resolução 466/12 sobre pesquisas desenvolvidas com seres humanos.

## RESULTADOS

As análises dos resultados foram realizadas a partir das respostas ao questionário, ao qual 6.545 pessoas participaram, sendo que 5.595 (85,5%) eram cuidadores e 950 (14,5%) eram idosos usuários e, para melhor compreensão desses grupos distintos, eles foram analisados separadamente.

No grupo dos idosos usuários do PMC, verificou-se que a maioria residia na região Sudeste (76,2%), era do sexo feminino (59,3%), com idade entre 60 e 70 anos (52,5%) e apresentaram uma renda familiar de até dois salários mínimos (85,9%). O idoso mais novo tinha 60 anos e o mais velho tinha 99 anos de idade, com mediana de 70 anos. Já no grupo dos cuidadores, verificou-se que a maioria residia na região Sudeste (72%), cuidavam de idosos do sexo feminino (59,1%), esses idosos tinham idade entre 60 e 81 anos (53%), sendo a idade do idoso mais novo de 60 anos e a do mais velho de 109 anos, com mediana de 81 anos, e que possuíam renda familiar de um a dois salários mínimos (74,5%). Os cuidadores apresentaram ensino médio completo ou incompleto (40,2%) e foram classificados como cuidadores informais (97,1%).

Em relação à descrição do PMC, no grupo dos idosos, verificou-se que a maioria sabia que era um programa do SUS (73,1%). Além da equipe do PMC também eram acompanhados pela equipe de saúde da família (58,3%), levaram até três dias para começarem a ser atendidos pelo programa (34,7%), recebiam visitas da equipe diariamente ou semanalmente (63,9%), conseguiam realizar exames e consultas com especialistas todas as vezes que foram solicitados pelos médicos (55,5% e 40,5%), recebiam visitas da equipe fora do horário de agendamento quando era necessário (53,9%), e sempre entendiam as orientações que eram passadas pelos profissionais da equipe sobre os seus cuidados (84,1%).

No grupo dos cuidadores, para esse mesmo item, descrição do PMC, verificou-se que a maioria sabia que era um programa do SUS (85,6%), além da equipe do PMC também eram acompanhados pela equipe de saúde da família (53,6%), levaram de três a sete dias para começarem a ser atendidos pelo programa (27,4%), recebiam visitas da equipe semanalmente (46,6%), conseguiam realizar exames e consultas com especialistas para os idosos todas as vezes que foram solicitados pelos médicos (58,3% e 40,3%), recebiam visitas da equipe fora do horário de agendamento quando era necessário (60,2%), sempre entendiam as orientações que eram passadas pelos profissionais da equipe sobre os seus cuidados (90,6%) e não foram convidados ou participaram de cursos para cuidadores (80,2%).

Para verificar a prevalência da satisfação, tanto de idosos quanto de cuidadores, foram testados cinco modelos na análise de classes latentes (ACL). O modelo com maior parcimônia foi o que possuía três classes, tanto para cuidadores quanto para os idosos, pois apresentaram valor elevado de entropia juntamente com valores significantes de LRT, além de ter apresentado menores valores de BIC e AIC.

A nomenclatura das classes foi atribuída de acordo com os padrões de respostas. A classe nomeada como “satisfeito” apresentou respostas positivas em todas as perguntas. A classe “moderadamente satisfeito” apresentou padrão misto de respostas na qual os participantes estavam satisfeitos com o serviço e o recomendariam a terceiros, mas acreditavam que algo ainda podia ser melhorado e avaliavam o serviço como regular. A classe nomeada como “insatisfeito” apresentou respostas negativas a todas as perguntas. Para essa análise, a opção de resposta “não sabe/não respondeu” foi considerada como valor ausente. Sendo assim, as respostas de 26 idosos e 129 cuidadores foram consideradas ausentes. Tanto idosos quanto cuidadores mostraram-se, em sua maioria, satisfeitos com o PMC, representando 93,7% e 90,2%, respectivamente (Tabela 1).

**Tabela 1:** Frequências absolutas e relativas das classes e seus respectivos nomes na população de idosos e de cuidadores.

Classificação	Idosos n (%)	Cuidadores n (%)
Insatisfeito	25 (2,7)	194 (3,5)
Moderadamente satisfeito	33 (3,6)	340 (6,2)
Satisfeito	866 (93,7)	4.932 (90,2)

Ao realizar a análise simples em cada grupo observou-se algumas distinções. No grupo dos idosos, apenas as variáveis sexo, renda familiar e saber que o programa é do SUS, não foram eletivas para a análise múltipla (Tabela 2). Com a realização da análise múltipla, permaneceram associados à satisfação dos idosos o fato de serem acompanhados também por uma equipe de saúde da família, o menor tempo entre o encaminhamento e a primeira visita pelo PMC, a realização de exames quando havia solicitação médica e ter visitas fora do horário agendado quando fosse necessário (Tabela 3).

**Tabela 2:** Análise simples da satisfação dos idosos com relação às variáveis socioeconômicas, demográficas e relacionadas ao PMC.

Fatores socioeconômicos, demográficos e relacionados ao PMC	Satisfação dos idosos					
	Satisfeito			Moderadamente satisfeito		
	OR	IC95%	p	OR	IC95%	p
Sexo						
Feminino	1,33	0,60–2,96	0,477	1,42	0,49–4,06	0,513
Masculino	1,00			1,00		
Idade						
≤ mediana (70 anos)	0,89	0,40–1,98	0,776	0,51	0,17–1,46	0,212
> mediana (70 anos)	1,00			1,00		
Renda						
0 a 2 salários mínimos	0,81	0,23–2,74	0,736	1,27	0,23–6,93	0,780
Maior que 2 salários mínimos	1,00			1,00		
Saber que o PMC é do SUS						
Sim	1,57	0,68–362	0,281	1,50	0,49–4,59	0,478
Não	1,00			1,00		
Atendimento compartilhado com a equipe de saúde da família						
Sim	3,78	1,56–9,16	0,003	2,42	0,79–7,33	0,118
Não	1,00			1,00		
Tempo entre o encaminhamento e o recebimento da primeira visita						
Até 3 dias	17,36	3,66–82,37	< 0,001	3,21	0,47–21,80	0,232
De 3 a 7 dias	11,81	2,48–56,15	0,002	4,50	0,70–28,79	0,112
De 8 a 15 dias	6,28	1,64–23,99	0,007	1,28	0,19–8,43	0,793
De 16 a 30 dias	2,53	0,74–8,61	0,137	0,32	0,02–3,55	0,355
Mais que 30 dias	1,00			1,00		
Frequência de atendimentos						
Diariamente ou semanalmente (uma vez por semana ou mais)	14,59	4,36–48,77	< 0,001	3,85	0,85–17,32	0,078
1 vez ao mês	6,28	1,64–23,99	0,007	1,28	0,19–8,43	0,793
2 vezes ao mês	2,53	0,74–8,61	0,137	0,32	0,29–3,55	0,355
3 vezes ao mês	1,00			1,00		
Acesso a exames, quando solicitados						
Sim, todas as vezes	16,53	5,02–54,35	< 0,001	4,20	0,83–21,04	0,081
Sim, algumas vezes	2,64	0,90–7,73	0,075	2,40	0,52–11,10	0,260
22Não, não precisou realizar exames	6,36	1,92–21,03	0,002	2,10	0,38–11,58	0,395
Não, não conseguiu realizar nenhum exame	1,00			1,00		
Acesso a consulta com especialistas, quando solicitados						
Sim, todas as vezes	16,64	4,59–60,25	< 0,001	2,80	0,57–13,75	0,205
Sim, algumas vezes	3,72	1,16–11,95	0,027	2,70	0,63–11,46	0,178
Não, não precisou de consulta com especialista	6,75	2,46–18,53	< 0,001	1,20	0,29–4,90	0,800
Não, não conseguiu realizar nenhuma consulta	1,00			1,00		
Atendimento realizado pela equipe do PMC fora do horário de agendamento						
Sim	4,32	1,39–13,37	0,011	4,33	1,12–16,67	0,033
Não	0,14	0,05–0,36	< 0,001	0,40	0,08–1,89	0,252
Não houve necessidade	1,00			1,00		
Compreender as orientações sobre os cuidados, passadas pelos profissionais da equipe do PMC						
Sempre	10,16	2,04–50,45	0,005	0,94	0,13–6,54	0,954
Na maioria das vezes	2,88	0,52–15,94	0,224	1,11	0,14–8,68	0,920
Quase nunca	0,16	0,01–1,96	0,154	0,33	0,01–6,65	0,472
Nunca	1,00			1,00		

PMC: Programa Melhor em Casa; SUS: Sistema Único de Saúde; OR: *odds ratio*; IC95%: intervalo de confiança de 95%.

Nota: a categoria “insatisfeito” foi utilizada como referência para a análise.

**Tabela 3:** Análise múltipla da satisfação dos idosos com relação às variáveis socioeconômicas, demográficas e relacionadas ao PMC.

Fatores socioeconômicos, demográficos e relacionados ao PMC	Satisfação dos idosos					
	Satisfeito			Moderadamente satisfeito		
	OR	IC95%	p	OR	IC95%	p
Atendimento compartilhado com a equipe de saúde da família						
Sim	4,22	1,33–13,34	0,014	3,43	0,83–14,16	0,088
Não	1,00			1,00		
Tempo entre o encaminhamento e o recebimento da primeira visita						
Até 3 dias	10,20	1,95–53,23	0,006	2,07	0,28–15,41	0,474
De 3 a 7 dias	9,66	1,80–51,78	0,008	3,30	0,45–24,01	0,237
De 8 a 15 dias	6,45	1,44–28,75	0,014	1,26	0,17–9,24	0,820
De 16 a 30 dias	1,57	0,40–6,15	0,515	0,20	0,01–2,45	0,210
Mais que 30 dias	1,00			1,00		
Acesso a exames, quando solicitados						
Sim, todas as vezes	5,74	1,11–29,77	0,037	1,53	0,19–12,33	0,685
Sim, algumas vezes	0,56	0,11–2,77	0,479	0,44	0,05–3,48	0,441
Não, não precisou realizar exames	1,57	0,31–7,94	0,584	0,12	0,008–1,93	0,136
Não, não conseguiu realizar nenhum exame	1,00			1,00		
Atendimento realizado pela equipe do PMC fora do horário de agendamento						
Sim	4,96	1,23–19,89	0,024	8,09	1,52–43,05	0,014
Não	0,22	0,06–0,78	0,019	0,24	0,02–2,69	0,248
Não houve necessidade	1,00			1,00		

PMC: Programa Melhor em Casa; OR: *odds ratio*; IC95%: intervalo de confiança de 95%.

Nota: a categoria “insatisfeito” foi utilizada como referência para a análise.

Já na análise simples, realizada com os cuidadores, observou-se que apenas as variáveis sexo e idade do idoso, não foram eletivas para a análise múltipla (Tabela 4). Após a realização da análise múltipla, permaneceram associados à satisfação dos cuidadores: o menor tempo entre o encaminhamento e a primeira visita pelo PMC, a realização de exames e de consultas com especialistas quando havia solicitação médica, ter visitas fora do horário agendado quando fosse necessário e compreender as orientações que eram passadas pela equipe do programa (Tabela 5).

**Tabela 4:** Análise simples da satisfação dos cuidadores com relação às variáveis socioeconômicas, demográficas e relacionadas ao PMC.

Fatores socioeconômicos, demográficos e relacionados ao PMC	Satisfação dos cuidadores					
	Satisfeito			Moderadamente satisfeito		
	OR	IC95%	p	OR	IC95%	p
Sexo dos idosos						
Feminino	0,92	0,69–1,24	0,623	1,10	0,77–1,59	0,582
Masculino	1,00			1,00		
Idade dos idosos						
≤ mediana (81 anos)	0,91	0,68–122	0,565	1,17	0,82–1,67	0,382
> mediana (81 anos)	1,00			1,00		
Renda familiar do idoso						
0 a 1 salário mínimo	0,94	0,47–1,86	0,870	1,86	0,82–4,23	0,137
Entre 1 e 2 salários mínimos	1,23	0,85–1,76	0,257	1,58	0,99–2,52	0,055
Maior que 2 salários mínimos	1,00			1,00		
Escolaridade						
Alfabetizado	0,38	0,04–3,44	0,389	0,75	0,06–8,38	0,815
Ensino fundamental (completo ou incompleto)	0,29	0,04–2,11	0,223	0,38	0,04–3,35	0,387
Ensino médio (completo ou incompleto)	0,21	0,03–1,59	0,134	0,34	0,04–3,00	0,335
Ensino superior (completo ou incompleto)	0,21	0,02–1,59	0,134	0,31	0,03–2,78	0,297
Pós-Graduação	0,05	0,00–0,45	0,008	0,16	0,01–1,98	0,154
Analfabeto	1,00			1,00		
Grau de relação com o(a) paciente						
Informal	0,16	0,02–1,19	0,074	0,34	0,04–3,00	0,337
Formal	1,00			1,00		
Saber que o PMC é do SUS						
Sim	1,41	0,97–2,04	0,068	1,10	0,70–1,75	0,662
Não	1,00			1,00		
Atendimento compartilhado com a equipe de saúde da família						
Sim	2,24	1,66–3,03	< 0,001	1,23	0,85–1,78	0,258
Não	1,00			1,00		
Tempo entre o encaminhamento e o recebimento da primeira visita						
Até 3 dias	10,48	5,81–18,91	< 0,001	2,18	1,09–4,36	0,026
De 3 a 7 dias	9,11	5,27–15,75	< 0,001	2,24	1,18–4,25	0,013
De 8 a 15 dias	3,59	2,30–5,60	< 0,001	1,44	0,83–2,49	0,184
De 16 a 30 dias	1,59	1,04–2,42	0,032	1,04	0,61–1,77	0,880
Mais que 30 dias	1,00			1,00		
Frequência de atendimentos						
Diariamente	2,27	0,60–2,57	0,225	0,97	0,20–4,76	0,978
Semanalmente (uma vez por semana ou mais)	1,54	0,54–4,39	0,415	1,33	0,38–4,59	0,648
1 vez ao mês	0,33	0,12–0,93	0,036	0,58	0,17–1,96	0,384
2 vezes ao mês	0,62	0,21–1,80	0,385	0,74	0,21–2,64	0,650
3 vezes ao mês	1,00			1,00		
Acesso a exames, quando solicitados						
Sim, todas as vezes	21,04	13,95–31,73	< 0,001	4,78	2,85–8,03	< 0,001
Sim, algumas vezes	4,99	3,34–7,45	< 0,001	3,19	1,90–5,35	< 0,001
Não, não precisou realizar exames	6,04	3,95–9,24	< 0,001	3,34	1,94–5,74	< 0,001
Não, não conseguiu realizar nenhum exame	1,00			1,00		
Acesso a consulta com especialistas, quando solicitados						
Sim, todas as vezes	18,86	11,06–32,15	< 0,001	2,90	1,58–5,32	0,001
Sim, algumas vezes	3,01	1,96–4,62	< 0,001	1,28	0,76–2,17	0,344
Não, não precisou de consulta com especialista	3,89	2,75–5,50	< 0,001	1,22	0,80–1,88	0,350
Não, não conseguiu realizar nenhuma consulta	1,00			1,00		
Atendimento realizado pela equipe do PMC fora do horário de agendamento						
Sim	2,51	1,70–3,68	< 0,001	1,37	0,87–2,16	0,171
Não	0,09	0,06–0,13	< 0,001	0,51	0,33–0,79	0,003
Não houve necessidade	1,00			1,00		
Compreender as orientações sobre os cuidados, passadas pelos profissionais da equipe do PMC						
Sempre	52,94	29,13–96,19	< 0,001	6,38	3,01–13,52	< 0,001
Na maioria das vezes	25,96	12,64–53,31	< 0,001	6,35	2,62–15,39	< 0,001
Quase nunca e Nunca	1,00			1,00		
Convite e/ou participação de curso/grupo para cuidadores, ofertados pela equipe do PMC						
Sim	2,60	1,59–4,25	< 0,001	1,30	0,72–2,34	0,376
Não	1,00			1,00		

PMC: Programa Melhor em Casa; SUS: Sistema Único de Saúde; OR: *odds ratio*; IC95%: intervalo de confiança de 95%.

Nota: a categoria “insatisfeito” foi utilizada como referência para a análise.

**Tabela 5:** Análise múltipla da satisfação dos cuidadores com relação às variáveis socioeconômicas, demográficas e relacionadas ao PMC.

Fatores socioeconômicos, demográficos e relacionados ao PMC	Satisfação dos cuidadores					
	Satisfeito			Moderadamente satisfeito		
	OR	IC95%	p	OR	IC95%	p
Tempo entre o encaminhamento e o recebimento da primeira visita						
Até 3 dias	5,84	2,91–11,75	< 0,001	1,76	0,81–3,79	0,149
De 3 a 7 dias	5,40	2,88–10,12	< 0,001	1,67	0,83–3,37	0,146
De 8 a 15 dias	3,28	1,87–5,76	< 0,001	1,40	0,75–2,63	0,282
De 16 a 30 dias	1,18	0,71–1,99	0,510	0,81	0,45–1,45	0,481
Mais que 30 dias	1,00			1,00		
Acesso a exames, quando solicitados						
Sim, todas as vezes	7,41	4,17–13,14	< 0,001	4,31	2,22–8,37	< 0,001
Sim, algumas vezes	2,97	1,74–5,06	< 0,001	2,79	1,50–5,18	0,001
Não, não precisou realizar exames	2,83	1,55–5,14	0,001	3,58	1,79–7,16	< 0,001
Não, não conseguiu realizar nenhum exame	1,00			1,00		
Acesso a consulta com especialistas, quando solicitados						
Sim, todas as vezes	6,02	2,89–12,55	< 0,001	1,72	0,78–3,78	0,176
Sim, algumas vezes	1,32	0,75–2,31	0,330	0,68	0,36–1,27	0,231
Não, não precisou de consulta com especialista	1,68	1,01–2,80	0,044	0,63	0,36–1,13	0,126
Não, não conseguiu realizar nenhuma consulta	1,00			1,00		
Atendimento realizado pela equipe do PMC fora do horário de agendamento						
Sim	1,81	1,12–2,91	0,015	1,21	0,70–2,07	0,488
Não	0,14	0,09–0,24	< 0,001	0,67	0,39–1,14	0,142
Não houve necessidade	1,00			1,00		
Compreender as orientações sobre os cuidados, passadas pelos profissionais da equipe do PMC						
Sempre	10,61	4,27–26,37	< 0,001	3,16	1,31–7,64	0,010
Na maioria das vezes	7,01	2,47–19,85	< 0,001	3,49	1,24–9,85	0,018
Quase nunca e Nunca	1,00				1,00	

PMC: Programa Melhor em Casa; OR: *odds ratio*; IC95%: intervalo de confiança de 95%.

Nota: a categoria “insatisfeito” foi utilizada como referência para a análise.

Ao analisar as variáveis independentes que apresentaram associação com o grupo de idosos e de cuidadores, nota-se que os resultados foram parecidos, ou seja, fatores praticamente iguais parecem influenciar na satisfação e nenhum deles estava relacionado aos fatores socioeconômicos e demográficos. Todavia, a variável região não foi colocada para análise nas regressões, uma vez que não apresentou número suficiente de indivíduos nos subgrupos para que a análise fosse realizada. Havia uma grande concentração de idosos e cuidadores satisfeitos e que residiam no Sudeste.

## DISCUSSÃO

Notou-se um perfil de cuidadores e idosos atendidos pelo Programa Melhor em Casa semelhante a outros programas da atenção domiciliar, no que diz respeito às características socioeconômicas e demográficas^12,13^. O PMC analisado por esta pesquisa apresentou algumas características que corroboram o que é preconizado pela legislação do programa e outras não apresentaram semelhança^14^.

No que se refere à satisfação, o presente estudo encontrou níveis elevados, tanto no grupo dos idosos (93,7%) quanto no dos cuidadores (90,2%). Estudos, frequentemente, têm apresentado níveis altos de satisfação dos usuários, tanto no âmbito internacional como no brasil, e isto ocorre independente da característica do serviço e da metodologia utilizada^15^. Alguns pesquisadores atribuem esse fato ao viés da gratidão, em que existe uma relutância em expressar opiniões negativas^18^ e normas de cortesia, como obrigações sociais em demonstrar respeito pelas autoridades ou entender a crítica como algo que mostra inconveniência social também podem estar associados à satisfação elevada^19^.

Entretanto, vale ressaltar a importância de se avaliar a satisfação de um serviço de saúde como forma de subsídio para o planejamento e retorno das ações implantadas, além de empoderar o usuário dentro da construção do serviço de saúde. Ademais, na tentativa de reduzir algum viés, o presente estudo avaliou a satisfação por meio de análise de classes latentes, pois levou em consideração a sua complexidade e a dificuldade em se medir ou mensurar fenômenos como a satisfação. Assim, a satisfação dos idosos e cuidadores com o PMC também pode ser justificada pela qualidade dos serviços ofertados, uma vez que em sua caracterização o programa apresentou indicadores positivos em praticamente todos os aspectos avaliados.

Também foram encontrados fatores que estão associados a essa satisfação elevada, tanto dos idosos quanto dos cuidadores. Com relação a esses fatores, o grupo dos idosos e dos cuidadores apresentou resultados semelhantes após a análise múltipla.

Martins et al.^20^ (2014), relatam que segundo a percepção dos usuários, os fatores que podem garantir uma melhor qualidade dos serviços públicos de saúde, em todas as complexidades, incluem diminuir o tempo de espera e a facilidade para agendar consulta em serviço especializado, corroborando os resultados deste estudo. A qualidade aferida pela percepção do usuário baseia-se no conceito que o usuário tem em relação ao fato de um serviço responder ou não às necessidades de cuidado^17,21^. Ou seja, os usuários realizam comparações entre o desempenho dos serviços e suas expectativas. Os resultados são bons quando a qualidade percebida é obtida^22,23^.

Neste estudo, o tempo de espera para iniciar os atendimentos do PMC foi classificado como pequeno, chegando em sua maioria a no máximo sete dias no grupo dos cuidadores e com, aproximadamente, seis vezes mais chance de estarem satisfeitos com o serviço se esse tempo fosse alcançado. No grupo de idosos esse tempo chegou a três dias, aumentando em aproximadamente 10 vezes a chance de satisfação com o serviço. Pode-se inferir que a expectativa foi alcançada e consequentemente o desempenho do serviço e sua qualidade obtiveram bons resultados, deixando os idosos e os cuidadores satisfeitos com o programa.

Com relação à garantia de acesso a serviços de diferentes complexidades, foram criadas redes de atenção à saúde que fornecem a possibilidade de uma atenção contínua e integral aos usuários^24^. O serviço de atenção domiciliar faz parte da rede de atenção à saúde e tem como objetivo articular-se com outros pontos de atenção à saúde, principalmente hospitais, serviços de urgência e com a atenção básica, buscando evitar demanda direta dos usuários. Ele também deve assegurar fluxos de encaminhamentos para especialidades e para métodos complementares de diagnóstico, tanto para situações eletivas quanto de urgências^7^. Como muitas vezes as equipes estão inseridas em hospitais ou em unidades de pronto atendimento, a facilidade do acesso de seus usuários a outros serviços pode aumentar, o que não ocorre em outros serviços públicos de saúde^25^.

Dessa forma, se justifica o fato de os idosos e de os cuidadores terem conseguido o acesso, na maioria das vezes, sempre que foram solicitados, mostrou relação significativa e levou a um aumento de aproximadamente até sete vezes a chance de estarem satisfeitos com o PMC.

As visitas fora do horário agendado também apresentaram associação significativa nos dois grupos (idosos e cuidadores), estando mais satisfeitos com o programa aqueles que recebiam essas visitas que, por sua vez, mostram uma disponibilidade maior da equipe para o cuidado. Estudo realizado por Gorina et al.^26^ (2014) analisou dois modelos de atenção domiciliar, em que a diferença entre eles estava no tempo disponibilizado pela equipe para o atendimento. O modelo no qual a equipe disponibilizava um tempo maior junto aos pacientes mostrou melhores níveis de satisfação por parte dos usuários, comparado ao outro modelo, no qual a equipe disponibilizava um menor tempo. Arruda e Bosi^25^ (2017) colocam a disponibilidade da equipe como uma oferta de ajuda, ressaltando a necessidade do estabelecimento de vínculo, que se apresenta como determinante no que diz respeito à satisfação dos usuários em relação ao atendimento.

No que tange à compreensão das orientações informadas pela equipe, estudo realizado por Kajonius e Kazemi^27^ (2016) encontrou forte associação entre a satisfação e o acesso a informações por parte dos usuários. Compartilhar informações os envolve em uma parceria ativa nos cuidados. Fatores como esse, que envolve o relacionamento entre equipe e usuários ou cuidadores, parece ser primordial na satisfação com o serviço. Neste estudo, compreender essas informações/orientações mostrou-se um forte fator entre os cuidadores na influência quanto a satisfação, levando a uma chance 10 vezes maior de satisfação entre aqueles que sempre compreendiam quando comparados com aqueles que nunca ou quase nunca compreendiam.

Por fim, o idoso ser acompanhado pelo PMC e pela equipe de saúde da família foi associado à satisfação, ou seja, aqueles que tinham seus cuidados compartilhados estavam mais satisfeitos. O compartilhamento do cuidado está relacionado à compreensão da equipe sobre as necessidades do paciente e à sensibilidade às singularidades de cada usuário. Promover esse cuidado compartilhado com as equipes ou serviços que compõem a rede é essencial, potencializa a eficácia e a eficiência da rede de atenção à saúde e, como a gestão do cuidado ao paciente da atenção domiciliar é complexa, existe uma exigência de integração de grande parte da rede^28^. Possivelmente, dentro dessa perspectiva de compartilhamento do cuidado, o idoso sente-se mais seguro e confortável com o atendimento e, consequentemente, isso reflete na percepção da qualidade do serviço e na sua satisfação.

Em suma, nos dois grupos (idosos e cuidadores) mais de 90% dos participantes demonstraram satisfação com o programa. E dentre os fatores investigados, nenhuma variável socioeconômica ou demográfica permaneceu sendo associada à satisfação. Enquanto as variáveis relacionadas ao programa, como: tempo entre indicação e primeira visita, acesso a exames e especialistas, visitas fora dos horários de agendamento, compreensão das orientações transmitidas pela equipe e ser acompanhado pelo PMC e pela equipe de Saúde da Família, mostraram associação estatisticamente significativa.

Nesse sentido, estudos como este possibilitam um melhor entendimento dos principais fatores relacionados à satisfação dos usuários e de seus cuidadores, auxiliando na compreensão acerca do programa e, também, podendo auxiliar no planejamento da gestão em saúde.
